# A report on ASIC2021: a conference on extracellular vesicle communication mechanisms

**DOI:** 10.20517/evcna.2022.31

**Published:** 2022-08-29

**Authors:** Ashley E. Russell, Susmita Sil, Shilpa Buch, Michael W. Graner

**Affiliations:** ^1^Department of Biology, School of Science, Penn State Erie, The Behrend College, Erie, PA 16563, USA.; ^2^Department of Pharmacology and Experimental Neuroscience, University of Nebraska Medical Center, Omaha, NE 68198, USA.; ^3^Department of Neurosurgery, University of Colorado Anschutz Medical Campus, Aurora, CO 80045, USA.

The **American Society for Intercellular Communication (ASIC) **held its first annual meeting, ASIC 2021, on October 21-23, 2021. This meeting brought together researchers from various disciplines and backgrounds to exchange ideas on various means of extracellular communication mechanisms, including tunneling nanotubules, extracellular vesicles (e.g., exosomes, microvesicles, midbody remnants, large vesicles/large oncosomes) and other circulating particles. Due to the ongoing COVID-19 pandemic, the meeting was held in a hybrid format. For those who were able, this afforded them the opportunity to attend in person, while others unable to travel were still able to participate in the meeting remotely via Zoom. Virtual participants were able to ask questions to presenters either verbally or via chat, and those selected for talks could pre-record their presentations and participate in a live Q&A session afterwards.

Although ASIC is a new society and the first meeting was held during the COVID-19 pandemic, the meeting still attracted more than 130 participants and was supported by seven sponsors, including Izon, Particle Metrix, Nanotech, SBI System Biosciences, Nanoview, Purigen Biosystems, Ceres Nano, and *Extracellular Vesicles and Circulating Nucleic Acids* journal. The meeting spanned three days, had 58 talks (38 of which were given in-person), and had one poster session on the 2nd day of the conference.

**Day 1 of meeting:** The meeting began on October 21st with opening remarks from Drs. Julie Saugstad and Ursula Sandau.

The first session (Pre-program) was an NIH workshop chaired by Julie Saugstad and Ursula Sandau and focused on funding mechanisms that may be of interest to the meeting attendees. John Satterlee (National Institute on Drug Abuse, National Institutes of Health) began the session by describing various current funding opportunities available through NIDA for all levels (undergraduate through experienced principal investigators), as well as more specific, EV-related R01 and R21 opportunities (e.g., PAR-20-147/PAR-20-148) focusing on extracellular RNA (exRNA) relevant to substance use disorders or HIV. Additionally, Satterlee imparted important advice to the audience about grant writing, including the “10 Commandments for R01 Applications” and encouragement to subscribe to the NIH’s Weekly Funding Opportunities and Notices email.

Next, Jill Morris (National Institute of Neurological Disorders and Stroke, National Institutes of Health) presented numerous funding opportunities in translational research, focusing on the need to bridge the gap between basic and clinical research. For example, the Innovation Grants to Nurture Initial Translational Efforts (IGNITE Program) currently has three opportunities available for translational projects in their early phases (PAR-21-122; PAR-21-123; and PAR-21-124). Through the NIH Blueprint, which provides information about neuroscience research and training opportunities, there are several funding mechanisms to allow for later-stage translational collaborative research projects between academia and industry (PAR-21-163 and PAR-21-233). Additionally, the National Institute of Neurological Disorders and Stroke has a biomarker program with several opportunities for funding at various stages of biomarker discovery including identification and analytical and clinical validation (PAR-19-315; PAR-21-056; PAR-21-057; PAR-21-058; PAR-21-059).

Christine Happel (National Center for Advancing Translational Science, National Institutes of Health) spoke next, providing an overview of the progress the NIH Common Fund Extracellular RNA (exRNA) Communication Program has made since its inception. The main goal of this program is to accelerate research in the field of exRNA biology, and a substantial amount of progress has been made in this area, with numerous landmark publications in *Cell* stemming from these efforts. Stage 1 of this program concluded in 2018 and focused primarily on understanding exRNA biogenesis and function, and clinical uses of exRNAs. Stage 2 aims to understand exRNA carriers and technologies to isolate and characterize single EVs. Data coordination and analysis are at the forefront of both stages and have allowed for the creation of important resources like the exRNA Atlas, which can be found at https://exRNA.org. Happel also touched on the similarities between EVs and SARS-CoV-2 and the redeployment of funding opportunities for adapting single-EV isolation technologies for the isolation of SARS-CoV-2 particles through the Rapid Acceleration of Diagnostics (RADx) program.

The final presenter for this session, Elzafir Elsheikh (National Institute of Standards and Technology, National Institutes of Health), discussed the need for EV reference materials and the importance of acknowledging how different isolation and characterization methods yield different results. Each method used to study EVs has its own advantages and limitations and provides its unique data profile of EV sizing and quantification profiles. The utilization of standardized reference materials better allows for comparison across different separation and characterization techniques, the most common of which are dynamic light scattering, nanoparticle tracking analysis, fractionation and light scattering, resistive pulse sensing, and electron microscopy. From several different ATCC cancer cell lines, EVs were isolated and characterized via tangential flow filtration and revealed heterogeneity across the cell lines in terms of EV quantity, size distributions, proteomic profiling, and vesicle morphology, especially with vesicles visualized via cryoEM. Also noted were the detrimental effects of freeze-thaw cycles on EVs and the need for a large collaborative study to assess reference materials across laboratories, as developing reference materials for EVs is quite a challenge.

Next, Gagan Deep (Wake Forest University) kicked off a pre-program NIH workshop session also chaired by Julie Saugstad and Ursula Sandau, where he discussed recent work examining the effects of a ketogenic diet on mild cognitive impairment. In this work, L1CAM was utilized to pulldown neuron-derived EVs from blood plasma from eleven cognitively normal control patients and nine diagnosed with mild cognitive impairment before and after the implementation of a modified Mediterranean ketogenic diet (MMKD). Post-MMKD, MCI patients had notably lower expression of neuroinflammatory markers associated with neuron-derived EVs (NDE), including amyloid Aβ1-42, phospho-Tau, neurofilament light chain, NF-κB, oxidized proteins, and other neuroinflammatory markers. Further, this work identified that monocarboxylate transporter 2 (MCT2) associated with NDEs may predict responsiveness to MMKD intervention. 

Michael Bukrinsky (George Washington University) was the next speaker in this session and discussed recent work from his laboratory focusing on Nef-carrying EVs, which can promote the formation of inflammatory monocytes and macrophages. Many people living with HIV develop other diseases such as HIV-associated neurocognitive disorder (HAND), cardiovascular disease, and other metabolic diseases; chronic inflammation may alter cholesterol synthesis and mediate the development of these disorders in people living with HIV. Despite antiretroviral therapy (ART), Nef is still present on the surface of EVs and has been shown to stimulate cholesterol biosynthesis and increase the abundance of lipid raft-associated TLR4 and TREM1 in macrophages, similar to what is observed when macrophages are exposed to other inflammatory stimuli. These Nef-EVs also drive TNF-α and IGR1R signaling and result in large responses to LPS exposures. Together these effects may result in persistent inflammation even after the use of ART and result in HIV-associated co-morbidities.

The next talk from Anil Prasad (Harvard University) also focused on neuroinflammatory EVs within the context of HIV. As substance use disorders (SUD) and HIV infection appear to have interactive, additive, and synergistic effects on the immune system and neuropathogenesis, this work examined how cocaine use intersects with HAND. The use of cocaine has been shown to exacerbate HAND by inducing inflammation in the central nervous system (CNS), modulating the immune responses, enhancing HIV replication, and altering intracellular trafficking of HIV-containing EVs, preventing lysosomal degradation. Further, cocaine enhances the release of EVs from immune cells infected with HIV, potentially through the downregulation of BST-2 (tetherin). Additionally, these EVs have increased the expression of viral genes, increased infectivity of T-cells, and promoted the release of inflammatory cytokines from human brain microvascular endothelial cells.

Xiaoli Yu (University of Colorado, Anschutz) presented an interesting talk focusing on glioblastoma (GBM) and meningioma (MMA)-derived EVs from blood plasma. These EVs contain high levels of IgG and may mediate complement activation and cascade; in GBM patients, IgG levels are often elevated in plasma and have a unique proteomic profile compared to healthy controls. It was demonstrated that multiple immunoassays could characterize IgG antibodies in plasma and plasma EVs from patients with GBM, MMA, and controls. Higher levels of Fc heavy chain IgG antibodies and IgG1 subclass were shown to be present in GBM plasma and EVs compared to MMA. The IgG antibodies in both GBM plasma and EVs produced complement-dependent cytotoxicity in the neuroblastoma cell line SH-SY5Y used as a neuronal surrogate. The higher IgG levels in the plasma and EVs in GBM patients and the high capacity of cell killing suggested that GBM IgG antibodies could play an important role in tumor pathogenesis.

Elena Batrakova (University of North Carolina) next presented work focusing on different techniques for loading EVs with drugs, including incubation at room temperature with or without saponin, altering the pH, electroporation, freeze-thaw, sonication, and extrusion. Macrophage-derived EVs were loaded with various cargo which was protected and highly stable, and these vesicles were delivered to the brain in an *in vitro* model. It is thought that the adhesion molecules on macrophage EVs were enabling this. In other murine models, the route of administration is an important consideration when planning drug delivery experiments; in this work, IV, intrathecal, and intranasal administration of EVs all resulted in delivery to the brain. The parental cells from which EVs are derived also influence EV loading and delivery and must also be considered.

The next presenter was Norman Haughey (Johns Hopkins University), whose lab has recently shown that brain-derived EVs communicate inflammatory damage in the periphery. Alzheimer’s disease (AD) is thought to induce chronic inflammation of both the central and peripheral immune system, and EVs may participate in the deposition or clearance of Aβ. In a mouse model of AD, Aβ was not found to be abundant in plasma or neuronal-derived EVs; however, adoptive transfer of plasma EVs from AD mice into wild-type mice resulted in an acute cytokine response, and these EVs were present in multiple tissues, including the brain. Interestingly, these EVs were enriched with ATPase ATP1A1 and modulated reactive oxygen species levels.

Nicole Noren Hooten (National Institutes of Health/National Institute of Aging) gave the next talk focusing on the HANDLs study which examined differences in life expectancy across various neighborhoods in Baltimore, MD and the use of EVs as potential clinical markers of mortality.

David Greening (Baker Heart and Diabetes Institute, Australia) presented next on a new class of EVs released from colon cancer cells; midbody remnants. These vesicles carry mutated KRAS G12V, activate MAPK signaling, and can be immunoaffinity-purified from plasma. Proteomic profiling also revealed that EVs may be cell signaling regulators. Additionally, the proteomic profile of heart-derived EVs may reflect changes associated with adipose phenotype, and engineered EVs may be useful for delivering cargo specifically to the heart to aid in cardiac repair.

Next, Gurudutt Pendyala (University of Nebraska Medical Center) presented work on sex differences in nicotine addiction. With tobacco use, the primary addictive compound is nicotine and female rats have higher nicotine characteristics and self-administer nicotine more frequently than males. When nicotine enters the brain, it induces dopamine release, altering synaptic activity and brain-derived EVs (bdEVs), as bdEVs from nicotine-addicted females had different proteomic profiles than saline-exposed females. Interestingly, sex-specific bdEV profiles were also observed, which may lend insight into the varying molecular basis of addiction observed between males and females. Using novel technology, Single EV Analyses with Multiplexing (SEAM), his group also validated the identified sex-specific bdEV markers.

Switching gears, Hameeda Sultana (University of Tennessee Knoxville) presented work focusing on arthropod EVs, flaviviruses, and their cargo. Flaviviruses cause diseases like West Nile and Lyme, and are typically transmitted from the bite of an infected insect, like a tick or mosquito. Data presented demonstrated that EVs isolated from tick saliva modulate wound healing and repair by altering the expression of CXCL12 and IL-8 in human skin cells. These EVs alter the immune response in skin cells as well.

The last research presentation of this session was given by Sowmya Yelamanchili (University of Nebraska Medical Center), who shared recent work investigating the role of EVs in methamphetamine (meth) use disorder. Meth is a highly addictive drug, and relapse is a major problem in recovering addicts; as such, there is a need for therapeutic interventions to prevent relapse. In the brain, meth induces chronic inflammation via microglial activation and causes blebbing from neurons and microglia, which may impact EV biogenesis. To study this, both a Rhesus macaque and a rat self-administration animal model were used. In the frontal cortices of macaques exposed to meth, there was an increase in genes associated with EV biogenesis pathways, specifically endosomal sorting complexes required for transport (ESCRT). Protein expression in these brains suggests there may be an enhanced release of exomeres in meth-exposed brains. EV-associated miR-29a was increased in methamphetamine abuse and may be involved in both drug-seeking behaviors and relapse. Artificial “EV-miR-29a” exposure induced proinflammatory cytokine release from microglia, specifically TNF-α, IL6, and ILβ, and resulted in synaptodendritic injury in neurons. Therapeutic interventions, including the administration of anti-inflammatory drugs like Ibudilast or AV411 in a rat model, resulted in a reduction in inflammation and EV secretion. Additionally, miR-29a was upregulated in the plasma of both macaques and humans addicted to meth, so it may serve as a promising biomarker and warrants further investigation. 

The last speaker in the pre-program NIH workshop session was Roger Alexander (Extracellular RNA Communication Consortium; ERCC), who spoke about an online extracellular RNA (exRNA) course from exrna.org. 

The official opening of the conference began with the ASIC Meeting Session 1, chaired by Fatah Kashanchi and Meta Kuehn. The first presenter of this session was Nobel Laureate Randy Schekman (University of California, Berkeley), whose presentation focused on selective protein sorting into exosomes with a role in cell differentiation and as a possible tool in genome editing. This study showed the means to deliver Cas9 and a guide RNA (gRNA) enclosed within exosomes, a subclass of small EVs, as a vehicle for efficient and targeted gene editing. Biotinylated Cas9 was expressed in donor HEK cells bound noncovalently to an integral multivesicular body (MVB) membrane protein, CD63-streptavidin. Exosomes formed in the MVBs were thus enriched in Cas9 and a gRNA. These were isolated by buoyant density sedimentation, followed by incubation with reporter cells containing an integrated copy of N-luciferase; exosome delivery of Cas9 and the guide RNA should allow the expression of luciferase. However, exosomes containing a similar level of Cas9 elicited no more than a 50% increase above the basal luciferase expression. The same was true of the conditioned medium containing Cas9-exosomes and even of donor and acceptor cells incubated together, which was separated by a vesicle-permeable membrane in a transwell chamber. Thus, for these EVs, the functional uptake to promote gene expression was not observed as found for those isolated from differentiating neurons. In contrast, donor and acceptor cells cocultured to near confluence showed a 60-fold increase in luciferase expression. Transfer of Cas9 appears to be mediated by open-end membrane nanotubular connections, which is likely dependent on membrane fusion at the point of junction from the donor to the recipient cell. A molecular investigation of the requirements for this transfer may permit the development of an efficient means for targeted delivery of Cas9/gRNA. It also suggests that donor-recipient cell combinations may not be wholly generalized.

The next presentation was from Lance Liotta (George Mason University), who shared work on tumor-derived EVs. The data presented indicated that different EV fractions expressed different proteins; for example, PDL1 was only observed in the 100k × *g* pellets. Further, there are different proteins expressed between *ex vivo* and cell line-derived EVs. Breast cancer-derived lymphatic EVs had very high expression of autophagic markers, specifically mitophagy proteins (e.g., PINK1) and may be transferring these mitophagic proteins to other cells. 

Dolores Di Vizio (Cedars-Sinai Medical Center) gave the next presentation focusing on the role of large oncosomes (LOs) in the metastatic spread of cancer to bone marrow. Large oncosomes are a type of EV derived from amoeboid cancer cells ranging from 1-10 µm in diameter. These were separated from highly metastatic amoeboid prostate cancer and breast cancer cells, and were found to induce tumor progression via bone marrow mesenchymal stem cells (BM-MSCs). At the gene level, LOs induced expression of the interferon-gamma and alpha pathways in BM-MSCs. This LO-driven anti-viral response also induced neutrophil recruitment towards BM-MSCs; these neutrophils displayed an immunosuppressive phenotype that could presumably benefit the tumor.

Ryan Flynn (Harvard University) next presented an interesting talk on glycoRNAs, which may bridge the gap between glycobiology and RNA biology. Recent evidence shows that small RNAs can be modified with N-glycans and displayed on the surfaces of cells. Metabolic labeling with azido sugars can be used for detecting biotinylated glycan RNA reporters, and streptavidin can be used to capture these biotinylated glycoRNAs. Many of these are noncoding RNAs, and different RNAs are associated with varying glycan structures.

Yoel Sadovsky (University of Pittsburgh, Magee Women’s Research Institute) gave the next presentation on the role of EVs in fetal-maternal communication. Placental-derived EVs from the trophoblast cell layer mediate virus resistance via the C19MC miRNA cluster as they protect against viral replication. The biophysical properties of trophoblast-derived EVs were also assessed and found that they have higher levels of phosphatidylcholines than other types of EVs, and may affect membrane rigidity.

Next, Kevin Morris (Griffith University, Australia) presented work on EV-mediated epigenetic repression of HIV in the context of DNA methylation of the LTR-retrotransposons, and HIV-targeted therapies via zinc finger proteins. An HIV promoter-targeting Zinc Finger protein (ZFP-362) fused to active domains of DNA methyltransferase 3A was developed. Engineered EVs were loaded with RNAs encoding this protein and were found to specifically repress HIV replication in cells while keeping T-cells alive. In NSG mice, these EVs reduced HIV in both the brain and bone marrow.

Meta Kuehn (Duke University) gave the last presentation of the evening, sharing recent insights into the mechanism and functionality of bacterial EVs. Bacteria are ubiquitous, and their EVs may be vehicles for the export of complex products. They can be isolated with similar techniques used to isolate eukaryotic EVs; however, there are many additional variables at play; for example, are the vesicles derived from gram-positive or gram-negative bacteria; are the cargo enveloped or not; are the proteins expressed on the inner and outer membranes of these EVs? All these variables can make for different vesicles. The group has shown that bacterial EVs can interact with plant pathogens and serve a protective function.

**Day 2 of meeting:** The following morning, the meeting began with a session on EVs and Cancer, and EVs in the CNS, and was chaired by Michael Graner and Ashley Russell.

The first talk of this session was delivered by Phil Stahl (Washington University). This talk focused on the physiological roles of EVs in a microenvironment, such as signaling, seeding, decoys, or exchange, and also recapped his lab’s 1983 seminal paper on the loss of transferrin receptors from reticulocytes as they mature into red blood cells. The EV disposal hypothesis was also presented, which outlines that EVs are “garbage bags” releasing unwanted cellular debris, like protein aggregates, into the extracellular space; however, it is not known whether this is by default or by design in that these vesicles are meant to be discharged, not necessarily as “garbage”. This segued into the signaling hypothesis that EVs mediate intracellular communication, proposed by Raposo in 1996; this sparked a change in mindset regarding the function and purpose of EVs. Some proteins are present in EVs because they participate in the vesicle formation process, whereas others are present because they have other roles, such as cell signaling. Further, Lotvall and Ratajczak showed that EVs can transfer miRNAs and mRNAs, which further supported the hypothesis that EVs participate in cellular communication.

Katia Mallouf (Harvard) presented the next talk, which focused on the use of EVs as tools to monitor neurologic diseases. There is a need for techniques to trace brain cell behavior *in vivo *and monitor the status of cells in the living brain. E-NOMI is a tool designed to trace and pulldown EVs derived from brain cells *in vivo *and *in vitro*, as they can be pulled down with beads due to the FLAG-TAG expressed on them. In this work, engineered human neural progenitor cells were implanted in mice and E-NOMI EVs were pulled out of the mouse blood plasma with anti-FLAG-TAG magnetic beads. 

The next presentation was given by Lucia Languino (Thomas Jefferson University) and discussed the use of EVs for cancer therapy as drug delivery molecules containing a specific target receptor, peptide, or drug. For example, integrin αvβ6 was able to target prostate cancer cells with relative specificity. To make these EVs, cells can be transfected or electroporated with specific siRNAs, as this is much more efficient than electroporating the EVs themselves with siRNAs.

Jeff Franklin (Vanderbilt University) delivered a talk focusing on purification strategies to optimize vesicle yield and parse the heterogeneity. Methodologies discussed were differential centrifugation, Optiprep gradient and direct antibody capture, fluorescent activated vesicle sorting, and size exclusion chromatography (SEC)/FPLC, as well as the use of hollow fiber bioreactors to produce high concentrations of EVs and nanoparticles. Different EV subpopulations from cetuximab-resistant cells were able to promote cetuximab resistance in previously sensitive lines.

Antonio Chiocca (Brigham and Women’s Hospital) continued the discussion on heterogenicity. One of the major reasons cancer therapies fail is the heterogeneity within tumors, as different cells within a single tumor can contain varying mutations. Cancer EVs, and their inherent heterogeneity, further extend this concept with effects on cells of the tumor microenvironment. Within the tumor microenvironment, there are “enablers” that help cancer proliferate; EVs can also be enablers and mediate changes in recipient cells and contribute to tumor immune suppression. This was detailed in the reduced T cell activation when the immune cells were exposed to glioblastoma EVs possessing the PDL1 portion of the PD1/PDL1 immune checkpoint axis.

Bojan Losic (Ichan School of Medicine at Mount Sinai) gave a talk focused on unannotated small RNA clusters associated with circulating EVs in the detection of early-stage liver cancer. Data generated from three independent extracellular RNA (exRNA) cancer datasets from 375 patients, including longitudinal samples, were used for this study. Results showed that exRNA, small RNA clusters (smRCs) were dominated by uncharacterized, unannotated small RNA with a consensus sequence of 20 bp. An unannotated 3-smRC signature was significantly overexpressed in plasma exRNA of patients with hepatocellular carcinoma (HCC). An independent validation in a phase 2 biomarker case-control study revealed 86% sensitivity and 91% specificity for the detection of early HCC from controls at risk. The 3-smRC signature was independent of alpha-fetoprotein and a composite model yielded an increased AUC of 0.93. These findings lead to the prospect of a minimally invasive, blood-only, operator-independent clinical tool for HCC surveillance, thus highlighting the potential of unannotated smRCs for biomarker research in cancer.

Dilorom Sass (National Cancer Institute) shared work examining EV-associated cytokines in breast cancer, aging, and inflammation. An in-house Luminex assay capable of detecting 35 cytokines probed the surface of EVs from samples of patients with breast cancer. They found intriguing results indicating that EV-IL2 and EV-GM-CSF expression was different between low pain *vs*. high fatigue in older breast cancer patients, but this trend was not observed in younger patients. These data suggest there may be age-associated differences in cancer-related EV profiles.

The next session on EVs and the CNS began with Aleksander Milosavljevic (Baylor College of Medicine), who presented work focusing on the deconvolution of the exRNA Atlas, which revealed six exRNA cargo types. RNA-binding proteins (RBPs) have over one million distinct exRNA binding sites, and many RBPs appear to carry exRNA in both cell culture supernatants and blood plasma. Interestingly, RBPs may show a preference for specific clusters of RNA, which warrants further investigation. 

Julie Saugstad (Oregon Health and Science University) shared recent data outlining the use of miRNAs as biomarkers for AD. This was the first study to obtain CSF from living donors and assess miRNA expression profiles that may be relevant in full-blown AD, and determine if they are also altered during mild cognitive impairment (MCI) in conjunction with APOE4. This work identified five CSF miRNAs that had a downward trend over time for those with MCI, and several miRNAs were also identified as potential candidates from patient plasma. Interestingly, however, these miRNAs were not identified in brain-derived EVs isolated from blood plasma using L1CAM. The miRNAs in CSF EVs were able to distinguish AD from Parkinson’s Disease, and pathway analysis showed convincing convergence with neurodegenerative disorders.

Navneet Dogra (Ichan School of Medicine at Mount Sinai) next presented work examining the neuro-secretome. For these experiments, EVs were isolated from brain tissue following mild digestion and fractionation with SEC; the protein and nucleic acid of each fraction were assessed. The first six fractions were found not to contain proteins or nucleic acids, but fractions containing EVs - identified initially by markers ALIX and FLOT1 - have a high abundance of DNA and RNA. In later fractions, enrichment of proteins and nucleic acids is observed once again and is likely non-vesicular. The reproducibility of over 15 brain samples was quite high.

Ursula Sandau’s (Oregon Health and Science University) presentation focused on the use of CSF EVs as biomarkers for brain diseases. Proximity of the biofluid to the brain/CNS makes it an attractive biomarker source. However, it is difficult to obtain and has far fewer circulating materials than plasma. Thus, it requires special attention to handling and EV isolation. EVs were isolated from CSF by SEC, using resins of different pore sizes (35 nm and 70 nm). Using various analytical methods, the two different SEC columns differentially separated smaller *vs*. larger vesicles. Curiously, NCAM1 was not detected in CSF-EVs. RNA profiling showed that some miRNAs reside exclusively in EV or protein fractions, while others can be found in both fraction types.

The last speaker of this session was Tsuneya Ikezu (Mayo Clinic Florida), who presented work focusing on how cell type-specific EVs define disease-related protein networks associated with astrocyte activation in AD. His group generated human induced pluripotent stem cells (hiPSCs) and differentiated them into neuronal, astrocytic, oligodendrocytic, and microglial cell types. Proteomic profiles of EVs from these differentiated iPSC cells contained cell-type specific markers: excitatory neurons (ATP1A3, NCAM1); astrocytes (LRP1, ITGA6); microglia-like cells (ITGAM, CD300A); and oligodendrocyte-like cells (LAMP2, FTH1). There were also 16 pan-EV marker candidates, including integrins and annexins. Cell type-specific EV proteins could also be found when comparing their data to CSF EV proteomic datasets, which also held true for brain-derived EVs. Correlation networks and pathway analyses identified proteins in each cell subset EVs with co-expression in AD. It was shown that astrocyte-specific EV (ADEV) markers were most significantly associated with AD pathology and cognitive impairment, thereby underscoring the role of ADEVs in AD progression. The hub protein from this module, integrin-β1 (ITGB1), was elevated in ADEVs purified from total brain-derived EVs and associated with brain Aβ42 and tau load in independent cohorts. From this, it was found that astrocytes are likely in an activated state due to IL1B, and astrocytic AD EVs are enriched in ITGB1. This correlated with Aβ42 and phosphoTau, and these EVs enhance neuronal uptake via integrin signaling. Thus, this study provides a featured framework and rich resource for analyses of EV functions in neurodegenerative diseases in a cell type-specific manner.

The next session of the day was EVs, CNS, and Viral Infections, chaired by Ramin Hakami and Leonid Margolis. The first presenter was Jay Debnath (University of California, San Francisco), who presented work on the intersection of secretory autophagy and EVs. Using genetic and molecular tools, the group assessed the proteomic profile of conditioned cell culture media, which revealed numerous proteins, many of which were ribosomal binding proteins (RBPs). Mass spectrometry-based quantitative proteomics found > 200 new putative targets of autophagy-dependent secretion, with co-fractionation of LC3B interactors and EV markers. Interestingly, LC3 processing and lipidation are required for RNA-binding protein secretion. Entire autophagosomes are not being secreted from cells, but various components are. For example, if the endolysosomal pathway is blocked, autophagy cargo receptors are secreted in EVs. If both the lysosomal and Rab27a (EV) pathways are blocked, cargo receptors remain inside the cell, but are not degraded, indicating important roles for both pathways in autophagy and EV release. As lysosomal pathways are often impaired in viral infections and CNS diseases, these results may have important clinical implications.

Next, Shilpa Buch (University of Nebraska Medical Center) presented work focusing on how the HIV-1 protein Tat primes and activates microglial NLRP3 inflammasome leading to synapto-dendritic injury in neurons via exosomes. Even after anti-viral treatment, HIV-1 Tat is still present. Findings from her group showed that HIV-1 Tat can cause the activation of microglial NLRP3 inflammasome, and induce the release of IL1B, further exacerbating the inflammatory response. These are important contributors to neuroinflammation in HIV-associated neurological disorders (HAND). Microglia-derived EVs (MEVs) were found to carry NLRP3 and IL1β cargoes, which upon being taken up by neurons resulted in synapto-dendritic injury and increased excitatory currents. Intriguingly, silencing of microglial NLRP3 inhibited the MEV-mediated neuronal damage. The role of NLRP3 in inflammation is well known; however, the role of the same NLRP3 in neuronal damage is an interesting finding and will add to the multifaceted therapeutic potential of NLRP3 blockers in HAND.

The third speaker of this session was Michal Toborek (University of Miami Health System). It is known that aging individuals living with HIV have an accumulation of amyloid in their brains compared to healthy controls. Data from this presentation indicated that endothelium-derived EVs carry Aβ, and impact the differentiation of neural progenitor cells. HIV infection enhances transendothelial transfer of A across the blood-brain barrier. Endothelial cell-derived EVs can deliver A to neural progenitor cells, induce inflammation, and block their differentiation. Thus, the intersection of aging and EV-driven neuroinflammation in HIV patients can be viewed as another risk factor for developing HAND.

The next presentation was very topical, focusing on COVID-19 EVs. Navneet Dhillon (University of Kansas Medical Center) presented work focusing on the role of EVs in COVID-19 and vascular injury. EVs were isolated from blood collected from asymptomatic, moderate (not on O_2_), moderate (on O_2_), and severe COVID-19 patients and found that these EVs are taken up by human pulmonary microvascular endothelial cells. Further, EVs from critically ill COVID-19 patients were more numerous compared to those from other disease states; these EVs had high proinflammatory markers and induced pulmonary microvascular endothelial damage. These studies could implicate blood EVs in vascular damage associated with COVID-19, and those EVs may play roles in “long-haul” disease.

Ross Jacobson (Particle Metrix) wrapped up this session with a presentation on the capabilities of the ZetaView instrument. The instrument takes 11 measurements per sample and provides size, concentration, and zeta potential. Further, vesicles can also be tagged with fluorescent antibodies or stains, and the ZetaView’s fluorescence filters (up to 4 lasers) can provide information on EV subpopulations as well. This work demonstrates the capabilities of combined fluorescence labels and NTA particle recognition which the field has found difficult to obtain.

The third session of the day was EVs, Drug Abuse, and Other Viruses, chaired by Michal Toborek and Sergey Iordanskiy. The first presenter was Christie Fowler (University of California, Irvine), whose presentation focused on nicotine and vaping and their effects on the CNS. Nicotine directly activates choroid plexus cells of the brain; these cells make CSF, and they release and filter CSF-EVs. miR-204 was shown to be upregulated in these cells and their EVs in response to nicotine exposure. When nicotinic receptors were blocked, this phenomenon was inhibited both *in vitro *and *in vivo*. EVs were labeled and injected into a mouse model and observed being trafficked in CSF to the medial habenula cholinergic neurons. Interestingly, THC was also found to upregulate miR-204 expression and affect NCAM1 expression in the brains of male but not female rats.

Presenting for a second time was Ursula Sandau (Oregon Health and Science University). This talk was focused on the effects of methamphetamine on plasma EVs and their miRNA cargo. Meth has been shown to alter Dicer1 and AGO_2_ (miRNA processing components) and stimulate EV release from neurons. It is also known that miRNAs are altered in brain regions associated with meth addiction, raising the possibility of EVs as biomarkers in meth addiction. The work involved the Methamphetamine Research Center with a study consisting of over 200 patients’ samples with associated clinical interviews. Plasma EVs were isolated via SEC and subpopulations were measured by vesicle flow cytometry. Platelet EV concentrations were correlated with self-reported depressive measures in patients. Further, several microRNAs were correlated with lifetime exposure to meth, frequency of use, and age of addiction onset, and others were further correlated with anxiety and memory in patients with active meth use.

Next, Guoku Hu (University of Nebraska Medical Center) gave an interesting talk focusing on the effects of morphine use on astrocyte primary cilium development and morphine tolerance. Opioid tolerance is a risk factor in opioid overdose. Via Sonic Hedgehog pathways, morphine was found to induce ciliogenesis and increase primary ciliary length in astrocytes *in vitro*, which is driven by EV release. The working hypothesis is that increased astrocyte EV release induces primary ciliogenesis in neighboring recipient astrocytes, leading to increase opioid tolerance. miRNAs may play a role in this mechanism as knockdown of Dicer resulted in an attenuation of this effect. In mice, the inhibition of primary cilia formation with MLN4924 and inhibition of EV release with GW4896 prevents morphine tolerance. As the opioid epidemic continues, mechanisms to prevent tolerance, further addiction, and cognitive decline become paramount in our strategies for treatment.

Susmita Sil (University of Nebraska Medical Center) discussed the role of astrocyte-derived extracellular vesicles in morphine-induced synaptic degeneration. It is known that morphine induces amyloidosis in astrocytes, and this is regulated by HIF1A. Astrocytes release EVs with the amyloid cargo, impacting neurons. Her studies demonstrate how morphine can cause activation of the autophagy pathway while blocking the autophagic flux in lysosomes. This resulted in increased EV production from astrocytes carrying autophagy proteins, which upon uptake by neurons, culminated in synaptic degeneration. It was also shown that morphine-mediated dysregulated autophagy and EV biogenesis were regulated by astrocytic NMDA-NR1. This study underscores the role of autophagy cargoes in morphine driven-astrocyte EVs mediated neuronal injury and synaptic alterations. Understanding how morphine hijacks the autophagy machinery to regulate EV release via the astrocytic NMDA-NR1, and how this phenomenon is involved with neurodegeneration is a novel concept, which can set the groundwork for future development of therapeutics for opiate addicts.

The next presenter was Sergey Iordanskiy (Uniformed Services University), whose presentation focused on how the bystander effect of ionizing radiation was mediated by endogenous retroviruses. It was shown that gamma radiation induced monocytes to differentiate into complex macrophage phenotypes. These macrophages express type I interferons (IFN-I) along with both pro- and anti-inflammatory cytokines via JAK/STAT pathways. These changes correlated with significantly upregulated expression of 622 viral retroelements, particularly from several clades of human endogenous retroviruses (HERVs). This study identified in irradiated macrophages an increased amount of the double-stranded RNA receptors, MDA-5 and TLR3, bound to an equivalent number of copies of sense and antisense chains of viral HML-2 RNA. This binding triggered MAVS-associated signaling pathways, resulting in increased expression of IFN-I and NF-κB-dependent inflammation-related genes. Knockdown of the HML-2 env gene was accompanied with downregulation of various HERV clades, suggesting, in turn, the dependence of retroviral expression on an interferon signaling that activates transcription of ERV elements whose promoter regions contain interferon regulatory factor- and NF-κB-binding sites. Silencing of HERV expression led to dramatically reduced expression and secretion of IFNα, proinflammatory and anti-inflammatory modulators in irradiated macrophages. Exposure of non-irradiated reporter THP1 cells with the culture media from HERV-silenced macrophages led to remarkable downregulation of expression of NF-κB-dependent proinflammatory genes and interferon-stimulated genes. Taken together, these data indicated that radiation stress-induced HERV expression enhances IFN-I and cytokine response, and results in increased levels of proinflammatory modulators released by macrophages, which is a potential inflammation-inducing mechanism in non-irradiated bystander cells.

Pooja Jain (Drexel University College of Medicine) next presented work focusing on the B and T lymphocyte attenuator (BTLA) protein, which can act as both an activator and inhibitor of B and T cell responses in the context of HTLV-1 chronic illness and inflammatory disease. HTLV-1 causes 80 different clinical syndromes and two main diseases, adult T-cell leukemia/lymphoma and HTLV-1-associated myelopathy. HTLV-1 infected cells released high quantities of BTLA, possibly in the form of EVs. In a treatment scenario, anti-viral therapies reduced levels of various immune checkpoint inhibitors in infected cells, including BTLA (seen in this context as a checkpoint inhibitor). There may be a potential role of EVs mediating these diseases as soluble and EV-associated PD-1 was observed in cell culture experiments.

Eva Poveda (Galicia Sur Health Research Institute) was the last presenter for this session and gave a talk focusing on cytokine profiles of EVs in HIV elite controllers a rare subset of people living with HIV that spontaneously control HIV replication without antiretroviral therapy (ART). Despite the rarity of these patients, the group was able to gather samples from 120 subjects, including 20 control subjects. Cytokine profiles (39 cytokines in a multiplexed bead-based assay) were assessed in plasma and EVs of healthy controls, as well as ART-naïve, ART-exposed, persistent elite controllers (control HIV infection over time), and transient elite controllers (control HIV infection, then lose that control). Interestingly, higher levels of IL18 and TNF-α in both plasma and EVs were found in elite controllers *vs*. ART-exposed patients, and within elite controllers, cytokine profiles can distinguish between persistent and transient control.

After a packed day of both in-person and virtual talks, young investigators participating in the meeting were encouraged to attend a NIH Grant Writing Workshop led by Fatah Kashanchi (George Mason University). During this interactive workshop, Dr. Kashanchi provided information about different types of grants, templates for writing specific aims pages, and walked through an on-the-spot grant proposal crowd-sourced from workshop participants. Also stressed was the importance of preliminary data as grant reviewers need to appreciate the feasibility of the proposed work. This was followed by a poster session.

**Day 3 of meeting:** The last morning of the conference began with a session on Technology and Treatments, and was chaired by Yuntao Wu and Pooja Jain. The first presenter of this session was David Walt (Harvard University), who focused on new tools for EV isolation, purification, and characterization with the goal of measuring cellular pathologic events based on materials in biofluids. In complex biofluids, it is difficult to separate and differentiate EVs from other particles such as lipoproteins, protein aggregates, *etc*., so new technologies are needed. Digital ELISAs, such as the single molecule array (SIMOA), use one bead per well with the idea that EVs will bind to beads in a 1:1 ratio. These sensitive measures could combine with optimized SEC to determine parameters for yield or for purity. From this, it was found that L1CAM is soluble and not associated with EVs isolated from CSF or plasma via SEC; however, it was associated with EVs from iPSC-derived neurons. 

Emeli Chatterjee (Massachusetts General Hospital and Harvard Medical School) gave a talk focused on an organ-on-chip model to characterize extracellular vesicles as functional biomarkers in cardiorenal syndrome (CRS). Heart failure (HF) can lead to renal injury and vice versa, and CRS is noted for this interface. Like many areas of pathology, EVs in CRS could be involved in organ cross-talk, intercellular communication, biomarkers, and potential therapeutic roles. With the development of a kidney proximal tubule organ-on-a-chip, the group isolated EVs from healthy donors, from patients with HF but no CRS, and from patients with both HF and CRS. Key findings showed that EVs isolated from patients with heart failure (HF) and healthy controls followed by exposure on kidney proximal tubule-chips for 72h led to increased mRNA expression of neutrophil gelatinase-associated lipocalin (NGAL) and IL-18 in human glomerular endothelial cells and proximal tubule epithelial cells following treatment with EVs. The increase was higher in HF patients with Type 1 CRS compared with those without CRS. There was also upregulation of kidney injury molecule 1 (KIM1) mRNA and cystatin C protein, suggesting induction of damage and dysfunction by HF/CRS EVs. This study bridges the gap between *in vitro* and *in vivo* models offering new approaches to identify the role of plasma EVs as potential biomarkers and effectors for CRS.

Next, Ramin Hakami (George Mason University) presented an interesting talk on the development of a novel microfluidic assay for monitoring the effects of live EV exchange in a physiologically relevant 3D environment. Size effects were apparent, as liposomes of 70 and 250 nm could get through the Matrigel (the 250 nm liposomes were less so than the 70 nm ones), but 500 nm fluorescent beads could not cross the Matrigel. With this platform, cells housed in one chamber of the chip could be transfected to express GFP, and their GFP-containing EVs could traverse through a Matrigel channel to other cells and be taken up by them. 

Meredith Chambers (University of North Carolina) gave a talk on the use of direct stochastic optical reconstruction microscopy (dSTORM) for visualizing EVs. This super-resolution microscopy can distinguish between a protein tagged on the surface of an EV and the rest of the EV to allow for vesicular protein localization. Future work aims to optimize three-color 3D dSTORM to allow for the localization of two proteins on a single EV.

Ryan McNamara’s (University of North Carolina) talk was presented by Dirk Dittmer. He discussed EV-encased nucleic acids and their use as a scaffold for chemotherapeutic agents for targeted tumor delivery. Kaposi Sarcoma (KS) is a type of cancer that is very prevalent in sub-Saharan Africa and is the most common type of cancer that people living with HIV develop. KS is caused by infection with KS herpes virus (KSHV) and KSHV RNAs promote tumor growth. KSHV-EVs attract endothelial cells; however, if the EVs have been loaded with the chemotherapy drug doxorubicin (DOX), the recruited cells will die. DOX appears to be better contained within EVs than in liposomes (DOXL), which may be due to its interaction with miRNAs in EVs. Interestingly, when DICER is knocked out, over half of the DOX signal in EVs goes away; this is likely due to DOX binding to other small RNAs within EVs other than miRNAs.

Roger Alexander (ERCC) gave a presentation summarizing the April exRNA Data Analysis Workshop (available on YouTube) and provided an update on the exRNA Atlas. There is a major lack of universal reference profiles and a great deal of batch variation, so the ERCC wanted to create a systematic pipeline for researchers to follow. Currently, over 7700 samples from different diseases and sample types are profiled in the exRNA Atlas; small RNA seq data processed through the exRNA toolkit are stored here and researchers are encouraged to submit new data to the Atlas as well.

The final session on EVs, RNA and Therapeutics was chaired by Christie Fowler and Susmita Sil. Alissa Weaver (Vanderbilt University) highlighted the biogenesis of RNA-containing extracellular vesicles at endoplasmic reticulum (ER) membrane contact sites. VAP-A is an integral ER protein that establishes points of intracellular contact between the ER and other organelles. As RNA complexes may be associated with the ER, it was speculated that ER-endosomal/multivesicular body contacts may be sources of RNA loading for EVs. VAP-A knockdown cells exhibited reduced ER-endosome contacts. This study elucidated the number of small RNAs that were altered in VAP-A KD small- and large-EVs compared to control cells. Density gradient fractionation revealed that VAP-A regulated a select subpopulation of small EVs that were enriched with RNA and RBPs. Analysis of small and large EVs for lipid content revealed that VAP-A controlled the levels of ceramide and cholesterol, two lipids involved in EV biogenesis. Furthermore, KD of the VAP-A binding ceramide and cholesterol transporters CERT and ORP1L led to similar defects in EV biogenesis. This study uncovered a novel pathway of EV biogenesis that takes place at the ER membrane contact sites.

Louise Laurent (University of California, San Diego) next presented work on exRNAs as biomarkers for preeclampsia. Preeclampsia is a leading cause of fetal and maternal morbidity worldwide; it manifests in the 2nd trimester, but its origins are thought to exist in the 1st trimester. Thus, early-stage biomarkers could be important to identify patients at risk and establish treatment regimens. Using discovery and verification cohorts from plasma, small RNA sequencing revealed miRNAs that may be associated with preeclampsia, and miRNAs could be clustered based on the patient’s status of severe preeclampsia, moderate preeclampsia, hypertensive controls, and normal controls. Most of the miRNAs originated from the liver, placenta, platelets, and red blood cells, and importantly, the current study did not distinguish between vesicular and non-vesicular RNAs; only total plasma RNA was assessed.

Next, Sophie Anderlind (Penn State Erie, The Behrend College) presented research on the effects of cortisol on iron transport proteins and EV release in placental cells. This work demonstrated that exposure of placental trophoblast cell line, BeWo, to physiologically relevant concentrations of hydrocortisone, resulted in the alterations in the expression of iron transport proteins- Transferrin receptor 1, Ferroportin-1, and DMT-1. Additionally, using size exclusion chromatography, they separated EVs from the conditioned media to assess protein composition following exposure of the cells to hydrocortisone. Future work from their team is aimed at addressing the effects of cortisol exposure on iron transport proteins in EVs, which could influence how iron is delivered during fetal development.

Heather Branscome (ATCC) presented work on retroviral infection of human neurospheres and the use of stem cell EVs to repair cellular damage. In this work, iPS neurospheres were generated from stem cells and infected with HIV-1 as a proof-of-concept platform for studying the effects of antiretroviral therapy on the brain. Interestingly, EVs isolated from the conditioned cell culture media of the stem cells used to create the neurospheres were found to have angiogenic, anti-inflammatory, and antiapoptotic effects.

The last presenter for the day was Botai Xuan (Izon), who presented an overview of Izon’s instruments and products, and expressed hope for a standardized workflow in EV isolation.

During the closing ceremony, awards were presented for both poster and oral presentations. Three poster award winners were Sarah Al Sharif, Sebastian Molnar, and Yijun Zhou. Three awards were given for the best talks by junior presenters: Meredith Chambers, Sophie Anderlind and Navneet Dogra.

ASIC would like to thank the Organizing Committee for organizing this meeting, especially given the hybrid format that was necessary to allow for the greatest exchange of scientific ideas. Special thanks are also extended to Gwen Cox for her commitment to ensuring this meeting ran smoothly.

Fatah Kashanchi, George Mason University

Leonid Margolis, National Institute of Child Health and Human Development, National Institutes of Health

Julie Saugstad, Oregon Health & Science University

Meta Kuehn, Duke University

Michael Graner, University of Colorado Anschutz

Janusz Rak, McGill University

## DECLARATIONS

### Copyright

© The Author(s) 2022.

